# Clinical features and *ALDH5A1* gene findings in 13 Chinese cases with succinic semialdehyde dehydrogenase deficiency

**DOI:** 10.1186/s12920-024-01925-4

**Published:** 2024-06-11

**Authors:** Hui Dong, Xue Ma, Zhehui Chen, Huiting Zhang, Jinqing Song, Ying Jin, Mengqiu Li, Mei Lu, Ruxuan He, Yao Zhang, Yanling Yang

**Affiliations:** 1 Department of Pediatrics, Peking University First Hospital, Beijing, 100034 China; 2 Department of Pediatrics, Women and Children’s Hospital, School of Medicine, Xiamen University, Xiamen, 361003 China; 3 Department of Respiratory Medicine, Beijing Children’s Hospital, National Centre for Children’s Health, Capital Medical University, Beijing, 100045 China

**Keywords:** Succinic semialdehyde dehydrogenase deficiency, 4-hydroxybutyric acid, Γ-aminobutyric acid, *ALDH5A1* gene, Novel variants

## Abstract

**Background and aims:**

To investigate the clinical features, *ALDH5A1* gene variations, treatment, and prognosis of patients with succinic semialdehyde dehydrogenase (SSADH) deficiency.

**Materials and methods:**

This retrospective study evaluated the findings in 13 Chinese patients with SSADH deficiency admitted to the Pediatric Department of Peking University First Hospital from September 2013 to September 2023.

**Results:**

Thirteen patients (seven male and six female patients; two sibling sisters) had the symptoms aged from 1 month to 1 year. Their urine 4-hydroxybutyrate acid levels were elevated and were accompanied by mildly increased serum lactate levels. Brain magnetic resonance imaging (MRI) showed symmetric abnormal signals in both sides of the globus pallidus and other areas. All 13 patients had psychomotor retardation, with seven showing epileptic seizures. Among the 18 variants of the *ALDH5A1* gene identified in these 13 patients, six were previously reported, while 12 were novel variants. Among the 12 novel variants, three (c.85_116del, c.206_222dup, c.762C > G) were pathogenic variants; five (c.427delA, c.515G > A, c.637C > T, c.755G > T, c.1274T > C) were likely pathogenic; and the remaining four (c.454G > C, c.479C > T, c.1480G > A, c.1501G > C) were variants of uncertain significance. The patients received drugs such as L-carnitine, vigabatrin, and taurine, along with symptomatic treatment. Their urine 4-hydroxybutyric acid levels showed variable degrees of reduction.

**Conclusions:**

A cohort of 13 cases with early-onset SSADH deficiency was analyzed. Onset of symptoms occurred from 1 month to 1 year of age. Twelve novel variants of the *ALDH5A1* gene were identified.

## Highlights

A 13-case cohort of succinic semialdehyde dehydrogenase deficiency was analyzed.

Onset of symptoms occurred from 1 month to 1 year of age.

All patients showed brain damage and high urine 4-hydroxybutyrate acid levels.

Eighteen *ALDH5A1* gene variants, including 12 novel variants, were identified.

Prompt symptomatic treatment may improve the long-term prognosis of this condition.

## Background

Succinic semialdehyde dehydrogenase (SSADH) deficiency, also known as 4-hydroxybutyric aciduria (OMIM 271,980), is a rare autosomal recessive inherited disorder caused by pathogenic variants of the *ALDH5A1* gene (OMIM 610,045), which cause succinate semialdehyde dehydrogenase deficiency and aberrant metabolism of the neurotransmitter γ-aminobutyric acid (GABA). Patients with this disorder show excessive accumulation of GABA, 4-hydroxybutyric acid, and other metabolites in the urine, blood, and cerebrospinal fluid. The clinical manifestations of this disorder are complex and diverse, with brain damage being the most prominent symptom. The incidence of SSADH deficiency is unknown, and has been estimated to be 1/460,000 [1]. To date, only approximately 450 cases are known worldwide [2]. Shibata N et al. [3] reported four cases of SSADH deficiency in 377 patients with inherited metabolic diseases in Japan, one in 250 cases in Vietnam, and two in 216 patients in China.

Only a few cases of SSADH deficiency have been documented in mainland China [4, 5]. The present study, which describes the largest cohort of Chinese patients diagnosed with SSADH deficiency reported to date, aimed to investigate the clinical features, metabolic profiles, and diagnosis and treatment strategies for such patients. We present the findings for this cohort of 13 Chinese patients with SSADH deficiency along with information regarding 12 novel variants in their *ALDH5A1* gene, which will enrich the data and clinical knowledge regarding the *ALDH5A1* gene variant spectrum.

## Methods

### Patients

Our study was approved by the Hospital Institutional Ethics Committee in accordance with the Declaration of Helsinki. Written informed consent was obtained from the parents of each participant. The 13 patients with SSADH deficiency were from 12 non-consanguineous Chinese families and were admitted to Peking University First Hospital between September 2013 and September 2023.

### Routine examination

The clinical and laboratory data at onset and follow-up were collected. Data from routine physical examinations as well as complete blood count, liver and kidney function, serum lactic acid, blood gas, and brain magnetic resonance imaging (MRI) data were analyzed.

Blood amino acids and acylcarnitine profiles in dried blood spots were analyzed by liquid chromatography tandem mass spectrometry (API 3200, Triple Quad 4500; Applied Biosystems, CA, USA). ChemoView software was used to automatically calculate metabolite concentrations. Urine organic acid levels were determined using gas chromatography-mass spectrometry (GC-MS, GCMS-QP2010 plus; Shimadzu, Kyoto, Japan) [3].

### Genetic testing

Genomic DNA from peripheral blood samples was extracted and purified. Next-generation sequencing was conducted in five patients (patients 2, 7, and 11–13) and their parents, while whole-exon sequencing was performed for the others. The DNA was sequenced on Illumina Hiseq2500 (Illumina, San Diego, USA). Each variant was compared with 1000 Genomics, the ExAC database (http://exac.broadinstitute.org/), and the gnomAD database (http://gnomad.broadinstitute.org/). Purified DNA samples were sent to Euler Genomics (Beijing), Berry Genomics Corporation (Beijing), and GrandOmics (Beijing) for next-generation sequencing or whole-exon sequencing to screen variants in patients. Mutation Taster, PolyPhen-2, and SIFT were also referred to for in silico analysis of the variants to predict pathogenicity. The variants were interpreted according to the Human Gene Mutation Database (HGMD; http://www.hgmd.cf.ac.uk/ac/index.php) and Clinvar (https://www.ncbi.nlm.nih.gov/clinvar). The pathogenicity of the selected variants was assessed on the basis of the American College of Medical Genetics and Genomics (ACMG) guidelines [6]. The variants were classified as pathogenic, likely pathogenic, variants of uncertain significance (VUS), likely benign, or benign.

### Statistical analysis

The collected data was statistically analyzed using the SPSS Version 26 software (IBM Corp., Armonk, NY, US). Continuous variables are given as the median (range), and the categorical variables are presented as frequencies and percentages. Fisher accuracy test was performed to evaluate differences between the groups. *P* < 0.05 was considered as statistically significant.

## Results

### Clinical features and biochemical findings

The 13 patients included seven (53.8%) male and six (46.2%) female patients. Their age at onset ranged from 1 month to 1 year (median 6 months), and the age at diagnosis ranged from 4 months to 15 years (median 1 year). Two patients (Patients 7 and 13) were sibling sisters. The other 11 patients came from unrelated families (Table 1).

**Table 1:** Clinical and biochemical manifestations of 13 cases with succinic semialdehyde dehydrogenase deficiency

Patient	Sex	Age of onset	Age of diagnosis	Clinical features	Peak urine 4-hydroxybutyrate acid (mmol/mol Cr)	Main treatment	Outcome
1	M	1 m	10 m	Psychomotor retardation, seizures, hypertonia, sleep disturbance	11.7	L-carnitine, vigabatrin, baclofen, taurine	1y3m now, with seizures frequently and developmental delay
2	F	3 m	4 m	Psychomotor retardation, seizures, hypertonia, sleep disturbance	11.2	L-carnitine, vigabatrin, baclofen, clonazepam	No improvement, loss of follow up since 7 m
3	M	3 m	4 m	Psychomotor retardation, seizures, sleep disturbance	25.0	L-carnitine, vigabatrin, taurine	4y3m now, with clinical improvement
4	M	4 m	8 m	Psychomotor retardation, seizures, hypotonia, sleep disturbance, regression after fever	20.4	L-carnitine, vigabatrin, phenobarbital, ketogenic diet	2y now, with seizures frequently and developmental delay
5	F	5 m	4y	Psychomotor retardation, sleep disturbance	46.1	L-carnitine, taurine	5y2m now, with clinical improvement
6	F	6 m	1y	Psychomotor retardation, seizures, dysphagia, sleep disturbance, emotional disturbance	7.4	L-carnitine, vigabatrin, taurine	3y2m now, with seizure and clinical improvement
7	F	6 m	7 m	Psychomotor retardation, seizures	40.7	L-carnitine, vigabatrin, levetiracetam	3y now, with seizures frequently
8	F	8 m	8 m	Psychomotor retardation, dystonia, sleep disturbance	10.5	L-carnitine, taurine, baclofen	3y now, with clinical improvement
9	M	9 m	1y	Psychomotor retardation, hypotonia	1.7	L-carnitine	No improvement, loss of follow up since 1y2m
10	M	9 m	1y	Psychomotor retardation, hypotonia, sleep disturbance	3.0	L-carnitine, taurine	4y4m now, with clinical improvement
11	M	1y	3y	Psychomotor retardation, seizures, suspected autism	14.7	L-carnitine, vigabatrin, taurine	Died at 3y10m after acute infections and status epilepticus
12	M	1y	1y10m	Psychomotor retardation, hypotonia, sleep disturbance	24.8	L-carnitine, taurine	9y now, with clinical improvement
13	F	1y	15y	Psychomotor retardation, sleep disturbance, misdiagnosed as “schizophrenia” aged 14y	20.8	L-carnitine, taurine	15y now, with clinical improvement

Notes: M = male, F = female, y = year, m = months

All 13 patients were born at full term and experienced psychomotor retardation, including delayed head-raising, sitting, and standing up in comparison with their normal peers. One patient (Patient 4) completed a developmental assessment, which yielded a developmental quotient value of 50. Seven patients (53.8%) had epileptic seizures, including absent seizures, generalized tonic-clonic seizures, focal seizures and myoclonic seizures. Patient 11 experienced convulsions from 3 years of age, and died of status epilepticus triggered by fever at the age of 3 years and 10 months. Eight patients (61.5%) experienced language developmental delays, which mainly manifested as impairments in expressive language. Sleep disturbances were observed in 10 (76.9%) patients and were characterized by difficulties in falling asleep or frequent night-waking. Dystonia was found in seven patients (53.8%), which was characterized by hypotonia and/or paroxysmal hypertonia. Three patients (23.1%) had mental and psychological disorders, of which one (Patient 11) had autism spectrum disorder, one (Patient 13) presented with schizophrenia, and two (Patients 6 and 13) exhibited emotional disturbances.

**Fig. 1:**
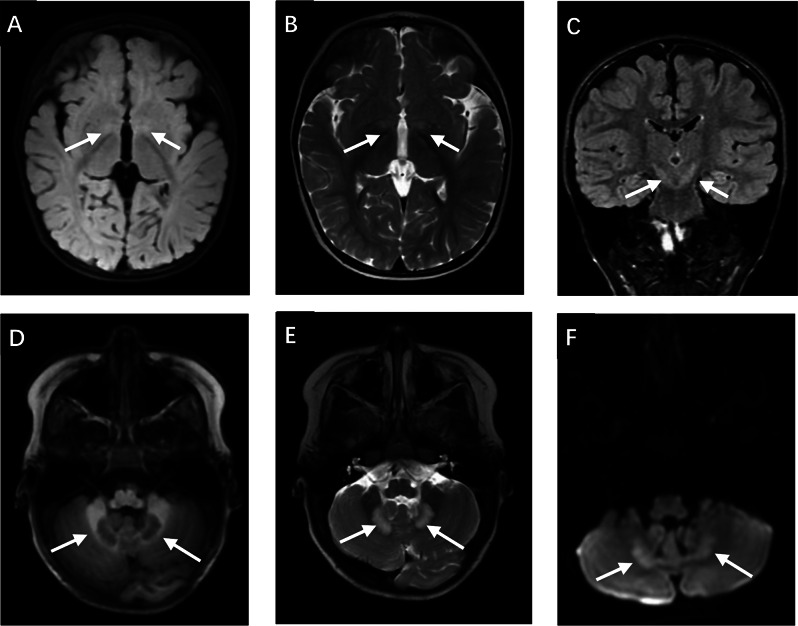
Brain MRI of Patient 4 (10 months old) showed symmetric T2 and T2-FLAIR long abnormal signals in both sides of the pallidus (**A**, **B**, **C**, the white arrow) and cerebellar hemispheres (**D**, **E**, **F**, the white arrow)

The liver and kidney functions as well as serum electrolyte levels of all 13 patients were normal. Ten patients underwent blood lactate tests, eight (80%) showed mildly elevated results (2.3–4.6 mmol/L; normal range, 0–2 mmol/L). Blood amino acid and acylcarnitine profiling showed no significant abnormalities. Urine 4-hydroxybutyrate acid levels were elevated (1.7-46.12 mmol/mol creatinine, not detected in the normal control). Eight patients underwent brain MRI, of which four (Patients 2–4 and 10) showed symmetric T2 and T2-fluid attenuated inversion recovery long abnormal signals in both sides of the pallidus (Fig. 1). One patient (Patient 6) showed bilateral abnormal signals in the cerebellar dentate nuclei, with mild atrophic changes in both cerebellar hemispheres. Two patients (Patients 8 and 9) showed cerebral atrophy and a widened extracerebral space. One patient (Patient 12) showed multiple lesions in the white matter. Electroencephalography was performed in six patients. One (Patient 4) showed increased background slow activity; one (Patient 2) showed suspected epileptic seizure; and six patients showed generalized spikes, spike-and-waves, and slow waves.

All 13 patients initially received L-carnitine (50–100 mg/kg/d), taurine (100–200 mg/kg/d), other symptomatic treatments, and rehabilitation training. Seven children with epileptic seizures were treated with vigabatrin (50–150 mg/kg/d), and two patients with frequent convulsions also received other anti-seizure medications. All patients were followed up for 3 months to 7 years. Anti-seizure treatment was effective in two patients (Patients 3 and 6), who showed a significant reduction in seizure frequency. Improvement in psychomotor development was observed in eight patients. One patient (Patient 11) aged 3 years 10 months died of status epilepticus after acute infections and fever. The urine organic acid profiles showed variable reductions in the 4-hydroxybutyric acid level in the children after treatment (1.24–6.6 mmol/mol creatinine).

### Gene analysis

A total of 18 variants were identified in the *ALDH5A1* gene of the 13 patients (Table 2). Six of these variants were reported to cause SSADH deficiency. Among these six variants, three were pathogenic [2, 4, 7], while other three were likely pathogenic variants [2, 5, 7, 8]. Twelve variants were novel, including eight missense variants (c.454G > C, c.479C > T, c.515G > A, c.637C > T, c.755G > T, c.1274T > C, c.1480G > A, c.1501G > C), one frameshift variant (c.427del), one nonsense variant (c.762C > G), one insertion (c.206_222dup), and one deletion (c.85_116del). Their pathogenicity was interpreted according to the standards and guidelines of the ACMG and the Association for Molecular Pathology (AMP) [6]. The eight novel missense variants were all in the highly conserved sites of the translated protein and were predicted as damaging variants by Mutation Taster, PolyPhen-2, and SIFT software. The missense variants were detected with extremely low or no frequency in the normal population and were not reported in the disease database. Among the 12 novel variants, three were pathogenic variants, five were likely pathogenic, and the remaining four were VUS, as shown in Table 2. The data have been uploaded to ClinVar (submission ID SUB13571749; ClinVar accession numbers, SCV003935160 to SCV003935171).

**Table 2:** The *ALDH5A1* gene variants and pathogenic analysis of 13 cases with SSADH deficiency

Patient	Allele	Extron (E) / Intron (I)	Nucleotide change^a^	Amino acid substitution	Reported	ACMG/AMP grading	ACMG classification	ClinVar accession number
1	Paternal	E4	c.617del	p.Phe206Serfs*5	[4]	P	PVS1 + PM2_Supporting + PM3	/
	Maternal	E1	c.206_222dup	p.Thr75Glyfs*22	This study	P	PVS1 + PM2_Supporting + PM3	SCV003935160
2	Paternal	E4	c.617del	p.Phe206Serfs*5	[4]	P	PVS1 + PM2_Supporting + PM3	/
	Maternal	E4	c.637C > T	p.Arg213Trp	This study	LP	PM2_Supporting + PM5 + PM3 + PP3	SCV003935164
3	Paternal	E3	c.479C > T	p.Ser160Phe	This study	VUS	PM1 + PM2_Supporting + PM3 + PP3	SCV003935165
	Maternal	E5	c.755G > T	p.Gly252Val	This study	LP	PM1 + PM2_Supporting + PM3_Supporting + PM5 + PP3	SCV003935166
4	Paternal	E2	c.427del	p.Thr143Qfs*8	This study	LP	PVS1 + PM2_Supporting	SCV003935167
	Maternal	E8	c.1274T > C	p.Leu425Pro	This study	LP	PM2_Supporting + PM3_Strong + PP3	SCV003935168
5	Paternal	E4	c.638G > T	p.Arg213Leu	[5]	LP	PM2_Supporting + PM3 + PM5 + PP3	/
	Maternal	E4	c.617del	p.Phe206Serfs*5	[4]	P	PVS1 + PM2_Supporting + PM3	/
6	Paternal	E10	c.1597G > A	p.Gly533Arg	[2, 8]	LP	PS3 + PM2_Supporting + PM3 + PP3	/
	Maternal	E10	c.1480G > A	p.Glu494Lys	This study	VUS	PM2_Supporting + PM3 + PP3	SCV003935169
7 and 13	Paternal	E3	c.454G > C	p.Glu152Gln	This study	VUS	PM2_Supporting + PM3 + PP3	SCV003935170
	Maternal	E10	c.1529C > T	p.Ser510Phe	[2, 7]	P	PS3 + PM2_Supporting + PM3_Strong + PP3	/
8	Paternal	E4	c.691G > A	p.Glu231Lys	[2, 7]	LP	PS3 + PM1 + PM3 + PM2_Supporting + PP3	/
	Maternal	E1	c.85_116del	p.Gly29fs	This study	P	PVS1 + PM2_Supporting + PM3	SCV003935171
9	Paternal	E3	c.515G > A	p.Arg172His	This study	LP	PM3_Strong + PM2_Supporting + PP3	SCV003935161
	Maternal	E10	c.1597G > A	p.Gly533Arg	[2, 8]	LP	PS3 + PM2_Supporting + PM3 + PP3	/
10	Paternal	E4	c.617del	p.Phe206Serfs*5	[4]	P	PVS1 + PM2_Supporting + PM3	/
	Maternal	I8	c.1344-2del	/	[7]	P	PVS1 + PM2_Supporting + PM3_Strong	/
11	Paternal	E10	c.1501G > C	p.Glu501Gln	This study	VUS	PM2_Supporting + PP3	SCV003935162
	Maternal	E8	c.1274T > C	p.Leu425Pro	This study	LP	PM2_Supporting + PM3_Strong + PP3	SCV003935168
12	Paternal	E5	c.762C > G	p.Tyr254*	This study	P	PVS1 + PM2_Supporting + PM3 + PP3	SCV003935163
	Maternal	I8	c.1344-2del	/	[6]	P	PVS1 + PM2_Supporting + PM3_Strong	/

^a^ The reference transcript is NM_001080.3, and the genomic build number employed was hg19 (human genome build 19)

ACMG/AMP = American College of Medical Genetics and Genomics and the Association for Molecular Pathology

P = pathogenic; LP = likely pathogenic; VUS = a variant of unknown significance

### Genotype-phenotype correlations

Four of the 13 patients (Patients 1, 2, 5 and 10) had c.617delT pathogenic variants, and the allele frequency of such variants was 15.4% (4/26). The four patients shared the same symptoms of psychomotor retardation and sleep disturbance (4/4, 100%), while the prevalence of sleep disturbances among the other patients without the c.617del variant was 66.7% (6/9), and there was no significant difference in the prevalence of these two groups (*p* = 0.497). Most of the variants in our study were located in exon 4 (7/26) and exon 10 (6/26) and were in the NAD^+^ binding domain of the gene. Notably, many of the variants in our study showed substitutions of Glu (5/26) and Gly residues (4/26).

## Discussion

This retrospective, single-center study of the largest reported cohort of Chinese patients with SSADH deficiency showed that the phenotypes and genotypes of the patients were complex. Our study identified 12 novel variants of the *ALDH5A1* gene. The onset age in Patient 1 was as early as 1 month of age. Patients 2 and 9 were lost to follow-up during the clinical course, while Patient 11 died of status epilepticus. The clinical-genetic correlations in our patients allowed us to speculate that the missense variants in Patients 2, 9, and 11 (c.637C > T, c.515G > A, c.1501G > C and c.1274T > C variants) all dramatically reduced SSADH enzyme activity and were crucial for disease severity. Drugs such as L-carnitine, vigabatrin, and taurine and rehabilitation training were all crucial for SSADH-deficient patients.

SSADH deficiency is a rare disorder of organic acid metabolism. SSADH participates in the metabolic processes of the inhibitory neurotransmitter GABA in the brain. SSADH enzyme deficiency blocks the transformation of succinic semialdehyde to succinic acid, causing the accumulation and significant increase in the levels of the intermediate metabolite 4-hydroxybutyric acid in the blood, urine, and cerebrospinal fluid, resulting in neurological damage [9]. Elevated GABA levels can disrupt the GABA shunt linking GABA transamination to the Krebs cycle, and maintaining the balance of excitatory-inhibitory neurotransmitters, while an increase in 4-hydroxybutyric acid levels is predicted to impact β-oxidation flux [9–11].

The clinical features of SSADH deficiency are highly heterogeneous, with neurological and psychiatric symptoms being the prominent manifestations of the disease [12–14]. Most patients experience mental retardation, hypotonia, hyporeflexia, and expressive language impairment in the first 2 years of life. Approximately 10% of patients present with progressive extrapyramidal symptoms and show neurological symptoms such as ataxia and dyskinesia [12]. The onset age was within 1 year for all 13 patients in this study, with developmental delay appearing as the initial symptom. The children exhibited varied forms of intellectual and motor retardation, and showed major motor and fine motor skills and language development severely behind their peers [12].

Epileptic seizures are the main neurological complications of SSADH deficiency. Approximately 50% of patients show epileptic seizures. Latzer et al. [14] reported that among 61 patients with SSADH deficiency, 30 (49%) showed epilepsy with onset age usually within 10 years (4 months to 19 years). The seizure types included absence seizures, generalized tonic-clonic seizures, myoclonic seizures, dystonic seizures, focal seizures, and epileptic spasms. A small number of patients also reported status epilepticus. The onset and severity of seizures were correlated with an age-related decline in the levels of GABA and GABA-related metabolites in the cerebrospinal fluid. The older the patients and the lower the concentration of metabolites, the more serious the epileptic seizures. In this study, seven children (53.8%) experienced epileptic seizures in various forms, including absence seizures, generalized tonic-clonic seizures, focal seizures, and myoclonic seizures. Cortez et al. reported that absence seizures were observed in SSADH-deficient mice and evolved into generalized convulsive seizures late in the third week of life, and that the underlying mechanism may involve inordinately high levels of 4-hydroxybutyric acid in the brain of the variant mice [1]. This seizure phenotype also manifested in human patients with SSADH deficiency. One patient died of status epilepticus, consistent with the findings of previous literature reviews. The onset of epileptic seizures in Patient 1 occurred at 1 month of age, which was younger than the age of onset reported in the previous literature. Thus, this study further broadened the age spectrum of the onset of seizures in patients with SSADH deficiency and provided more clinical evidence for epilepsy research in relation to this disease in the future.

Neuropsychiatric symptoms were also common manifestations in patients with SSADH deficiency [12, 13], with approximately 5–15% of adolescent and young adult patients showing these symptoms. Moreover, 63–70% of the patients have been reported to show attention deficit hyperactivity disorder, obsessive compulsive disorder, anxiety, autism-like behaviors, aggression, and sleep disturbances. In our study, 76.9% of the patients showed sleep disturbances and 23.1% showed psychiatric disorders. One patient had autism spectrum disorder, one showed schizophrenia, and 15.4% of the patients showed emotional disorders. These finding are consistent with the results of previous studies. Among these patients, Patient 13 was diagnosed as showing schizophrenia at the age of 14 years, indicating that hallucinations, delusions, and schizophrenic behaviors were more common in older children and adolescents. The results suggest that inherited metabolic disorders such as SSADH deficiency should be considered in the differential diagnosis of children with autism or schizophrenia accompanied by epileptic seizures and psychomotor retardation.

The key methods to detect SSADH deficiency are urine organic acid assays and gene analyses, with patients showing high levels of 4-hydroxybutyric acid in the urine as well as elevated concentrations of 4-hydroxybutyric acid and GABA in the serum and cerebrospinal fluid. Malaspina et al. [10] reported that the concentration of 4-hydroxybutyric acid in the cerebrospinal fluid of patients with SSADH deficiency ranged from 116 to 1110 µmol/L (normal reference range, < 3 µmol/L), with the GABA concentration ranging from 13.6 to 22.4 µmol/L (normal reference range, < 12 µmol/L). The patients’ serum lactate levels were mildly increased, while the blood amino acid and acylcarnitine profiles showed no specific abnormalities except for a mild decrease in free carnitine levels. A definite diagnosis of SSADH deficiency depends on the results of urine organic acid analysis, the SSADH activity test in lymphoblasts and skin fibroblasts, and *ALDH5A1* gene analysis [2, 3]. In this study, all patients showed an increase in urine 4-hydroxybutyric acid concentrations. Their serum lactate levels were also mildly elevated. These findings were consistent with the biochemical characteristics of SSADH deficiency.

The typical brain MRI abnormalities in SSADH-deficient patients include symmetric T2 signal prolongation on both sides of the globus pallidus, cerebellar dentate nucleus, subcortical white matter, and brainstem, and variable atrophy in the brain and cerebellum [16]. Afacan et al. [17]. reported that in patients with SSADH deficiency, the GABA levels in the regions described above were higher, the ratio of glutamate and glutamine/Cr was higher, the Cho/Cr ratio was not significantly abnormal, and the NAA/Cr ratio in the posterior cingulate was slightly lower than the corresponding values in the control group. In this study, all patients’ brain MRI scans showed different abnormalities, of which symmetrical T2 hyperintensity in the bilateral globus pallidus and cerebellar dentate nucleus were the main changes, followed by cerebral white matter lesions and brain atrophy, which was consistent with previous literature reports. Thus, these lesions may be related to the clinical symptoms of athetosis and ataxia in children. Interestingly, we observed that among four patients in our study who showed symmetric T2 and T2-FLAIR abnormal signals on both sides of the pallidus, two carried the pathogenic c.617del variant in the *ALDH5A1* gene. Therefore, we inferred that SSADH-deficient patients with the *ALDH5A1* c.617del variant may show a higher tendency of both pallidus abnormalities in brain MRI. However, because of the limited number of patients, more research is required to confirm the correlation between the phenotype severity and brain imaging findings.

However, symmetrical T2 hyperintensities at these sites could also be seen in patients with glutaric aciduria type 1 and other organic acidemia; thus, finding was not a specific change associated with SSADH deficiency and had no diagnostic significance. Children with similar brain MRI changes should undergo assessment of clinical symptoms, biochemical metabolic tests, and genetic analysis for further differential diagnosis.

**Fig. 2:**
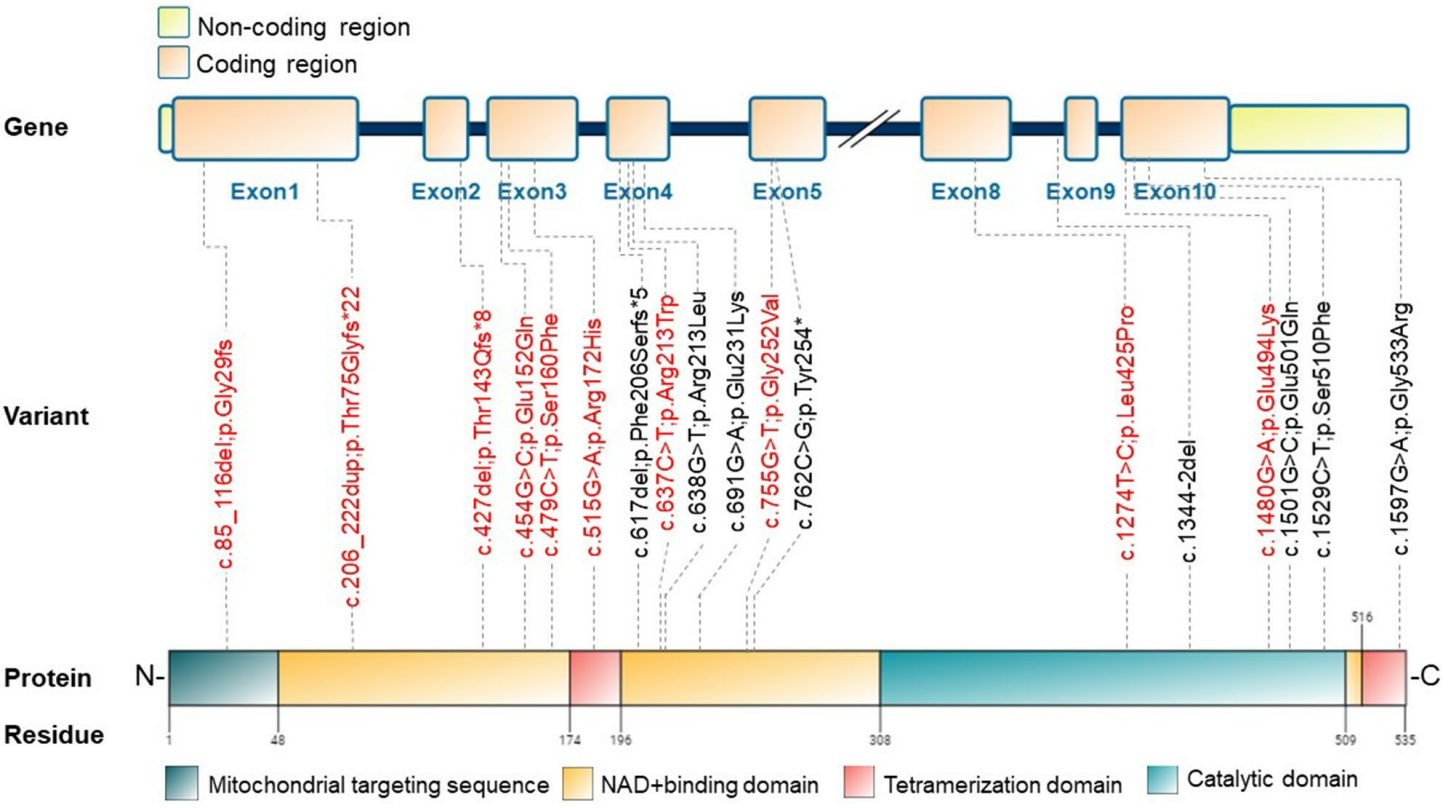
Exon and domain structure of *ALDH5A1*, and localization of *ALDH5A1* variants in this study. The human *ALDH5A1* gene exhibits 535 amino acids. The orange box shows the exons, joined by the dark blue lines representing introns. Colored blocks refer to the protein domains as indicated. Dashed lines connect the reported disease-causing variants with the respective exons and protein domains. The variants identified in our study are shown in red color

The *ALDH5A1* gene is located on chromosome 6p22.3, contains 10 exons, and encodes a polypeptide of 535 amino acids [18] (Fig. 2). More than 70 variants have been reported in the HGMD, with missense, deletion, insertion, and frameshift variants accounting for 57%, 17%, 9%, and 8%, respectively. At present, no hotspot mutations, or prevalent mutations of the *ALDH5A1* gene have been identified [4, 18, 19]. Four of the 13 patients in this study had c.617delT pathogenic variants, suggesting this variant may be common in the Chinese population. The four patients with the c.617del variant on *ALDH5A1* shared the same symptoms of psychomotor retardation and sleep disturbance. However, the patients in our study showed variable degrees of psychomotor retardation. The prevalence of sleep disturbances in patients with and without c.617del was 100% (4/4) and 60% (6/9), respectively, which indicated that the c.617del variant had some relationship with sleep disturbances, although there was no statistical difference between the two groups. However, the exact mechanism was still unclear, and should be investigated in future studies.

A total of 18 variants were detected in the *ALDH5A1* genes of these 13 patients, of which six were previously reported and 12 were novel. Notably, eight of the novel mutations are missense changes. Apart from eight missense variations, one each of frameshift, nonsense, insertion, and deletion variants were detected. On the basis of the ACMP/AMP pathogenicity grading, three variants were categorized as pathogenic, five as likely pathogenic, and four as VUS. These 12 novel variants further enriched the *ALDH5A1* pathogenic gene variant database. The reported literature showed no obvious genotype/phenotype correlations in the patients with SSADH deficiency. Gibson et al. reported that the disability and symptoms vary greatly even in the two siblings carrying the same pathogenic *ALDH5A1* variants [20].

The highly variable clinical presentations of the patients in our study also indicated the poor correlations of the genotype and phenotype of the disease. Our study did not include enzyme-activity analyses of the patients, so we attempted to analyze the genotype/phenotype correlations on the basis of the patients’ clinical manifestations. Most of the variants identified in our study were located in the NAD^+^-binding domains of the ALDH5A1 protein (10/18, 55.6%), while five other variants were located in the catalytic domain. Such variants may cause impairment of NAD^+^ binding and exhibit strongly negative effects on the catalytic activity of SSADH [21]. We speculated that the NAD^+^-binding domain and the catalytic domain were crucial sites of the enzyme. The variants in such sites would play vital roles in enzyme activity and impact the disease severity. Four variants in this study showed substitution of Gly residues to other amino acids. As Gly residues have been shown to be especially well-conserved in the ALDH family [22], such variants should show impairment of SSADH oligomerization along with disease-causing stability.

In Patient 2, who showed very severe disease course, c.637C > T (p.Arg213Trp) was identified among the null frameshift variants. The ACMG grading for this variant was likely pathogenic. This case provided a convincing proof for the association of severe enzyme activity deficiency with such missense mutations.

Patient 9 was also lost to follow-up probably because of the poor response to treatment. In this patient, c.515G > A (p.Arg172His) coexisted with a second allele (c.1597G > A), resulting in the substitution of a conserved glycine residue by arginine (p.Gly533Arg). This variant was estimated to be likely pathogenic, which strongly suggested that single amino acid variations would have severe effects. As for Patient 11, who died of status epilepticus, the presence of the variants c.1501G > C (p.Glu501Gln) and c.1274T > C (p.Leu425Pro) also suggested severe adverse effects on enzyme activity.

Curative treatments for SSADH deficiency are unavailable at present, and the existing clinical treatment regimens mainly focus on ameliorating some of the symptoms [23, 24]. Vigabatrin is an irreversible inhibitor of GABA transaminase that can inhibit the conversion of GABA to 4-hydroxybutyric acid, increase GABA levels in the serum and cerebrospinal fluid, and reduce the level of 4-hydroxybutyric acid in patients. High doses of vigabatrin can prolong the survival time of SSADH-deficient model mice [23–25]. Valproic acid may affect mitochondrial function and inhibit residual SSADH activity and should be avoided in the treatment of SSADH deficiency. Methylphenidate, risperidone, fluoxetine, and benzodiazepines can be used for children experiencing psychological symptoms such as anxiety, depression, and hallucinations [25]. In addition, a ketogenic diet [26], L-carnitine, and taurine have also been applied for the clinical treatment of this disease [2, 4].

At present, clinical trials are underway for drugs such as NCS-382 (4-hydroxybutyrate receptor antagonist), SGS-742 (GABA-B receptor antagonist) [27], and other therapeutic options targeting GABA and 4-hydroxybutyrate receptors, as well as mTOR inhibitors. Enzyme replacement therapy [28] and other therapeutic modalities have also shown some clinical effects [23]. These methods have indicated some potential as long-term treatment options. An experimental study on a mouse model suggested that glutamine supplementation improved peripheral but not central glutamine deficiency [29].

All 13 patients in this study received symptomatic treatment, including L-carnitine and nutrition support therapy. Seven patients with epilepsy were all treated with vigabatrin. During the follow-up period, two patients showed remission of convulsions, one was lost to follow-up, one died of status epilepticus, and three continued to experience frequent seizures. The symptoms related to intellectual and motor development, epileptic seizures, language development, and sleep disorders in eight patients were alleviated after treatment. During the follow-up period, all patients showed a reduction in the urine 4-hydroxybutyric acid level, indicating the effectiveness of the treatment.

As discussed above, most of the variants found in our study were located in the NAD^+^-binding domain, and therapeutic strategies such as enzyme replacement therapy or gene therapy may focus on the gene variants affecting such domains to achieve more precise treatment.

Our study had some limitations. One was the relatively small number of patients enrolled and short duration of follow up, which is understandable considering the ultra-rare nature of the disease. Another was that the genotype/phenotype correlation study was solely based on the clinical manifestations of the patients. The quantity and type of enzyme proteins, as well as their structural and functional impairments, contribute to phenotype severity in SSADH deficiency [5], but the retrospective nature of our study precluded evaluations of enzyme activity. Moreover, we could not analyze the protein structure alterations caused by the gene variants due to the limited laboratory equipment. We would enroll more patient in the further and try to conduct the studies focusing on the enzyme activity and protein structure caused by the variants. Our study also focused little on the precise treatment of the disease except vigabatrin. Future studies on the pathomechanisms and treatment of SSADH deficiency should aim to perform functional analysis and animal model investigations, along with prospective investigations to enroll more patients to obtain more scientific conclusions.

In conclusion, SSADH deficiency is an ultra-rare disease caused by an abnormal GABA metabolic pathway. The clinical symptoms of the patients are complex and diverse. Prominent manifestations include movement disorders, delayed language development, and neuropsychiatric symptoms. With advancements in urine organic acid analysis and genetic testing technology, the early diagnosis rate of SSADH deficiency has increased. For patients showing unexplained language retardation, epileptic seizures, and psychomotor symptoms, urine organic acid and genetic testing should be performed promptly to ensure early diagnosis and treatment and avoid misdiagnosis. As for the genotype/phenotype correlations in our patients, the findings indicated that c.637C > T, c.515G > A, c.1501G > C and c.1274T > C all dramatically reduce SSADH enzyme activity and are crucial for disease severity. Our study further enriched the research on *ALDH5A1* genotypes and clarified the clinical manifestations. Future studies should consider therapeutic strategies such as enzyme replacement therapy or gene therapy focusing on the gene variants affecting such domains.

## Data Availability

The datasets generated and/or analysed during the current study are available in the ClinVar repository. Accession Number SCV003935160 to SCV003935171.

## Abbreviations

ACMG: American College of Medical Genetics and Genomics
AMP: Association for Molecular Pathology
GABA: Gamma-aminobutyric acid
HGMD: Human gene mutation database
T2-FLAIR: T2-fluid attenuated inversion recovery
MRI: Magnetic resonance imaging
SSADH: Succinic semialdehyde dehydrogenase
VUS: Variants of uncertain significance
